# STEAP2-associated modulation of PI3K/AKT/mTOR signaling contributes to ginkgetin-induced apoptosis in bladder cancer cells

**DOI:** 10.1186/s41065-026-00686-7

**Published:** 2026-05-01

**Authors:** Pengze Wu, Lin Chen, Jin Yang, Zhengkang Liang, Xiaofeng Yin, Shaowen Zhou, Zhilin Deng, Yafei Yang

**Affiliations:** 1 North Sichuan Medical College, Nanchong, 637000 Sichuan China; 2 Department of Urology, Clinical Medical College & Affiliated Hospital of Chengdu University, Chengdu, China; 3 Zunyi Medical University, Zunyi, Guizhou, 563006 China; 4 Jianyang Traditional Chinese Medicine Hospital, Chengdu, Sichuan China; 5 Department of Urology, The Third Affiliated Hospital of Shenzhen University (Luohu Hospital Group), Shenzhen University, Shenzhen, China; 6 Department of Clinical Medicine, North Sichuan Medical College, Nanchong, 637000 Sichuan China

**Keywords:** Ginkgetin, Bladder cancer, PI3K/AKT/mTOR signaling pathway, STEAP2, Apoptosis

## Abstract

**Background:**

Bladder cancer remains a major urologic malignancy with substantial recurrence and progression risk, underscoring the need for mechanism-informed therapeutic candidates. Ginkgetin, a biflavonoid derived from *Ginkgo biloba* leaves, has shown antitumor potential in several cancer settings, yet its key signaling axis and actionable molecular node in bladder cancer have not been systematically defined.

**Methods:**

We evaluated ginkgetin across multiple bladder cancer cell lines (5637, T24, HT-1376, J82) and normal urothelial cells (SV-HUC-1) using viability assays and IC₅₀ estimation. Antitumor phenotypes were assessed by colony formation, wound-healing migration assays, EMT marker profiling, and Annexin V/PI flow cytometry. Network pharmacology and RNA-seq were integrated to prioritize enriched pathways, followed by western blot validation of PI3K/AKT/mTOR phosphorylation. An insulin reactivation (“rescue”) strategy was used to functionally test pathway dependence. Transcriptome-derived candidates were further examined by RT–qPCR and STEAP2 overexpression to probe node-level involvement. In addition, molecular docking and 100-ns molecular dynamics simulations were performed to characterize ligand–target binding stability.

**Results:**

Ginkgetin suppressed bladder cancer cell viability in a time- and dose-dependent manner at low micromolar concentrations, while normal urothelial cells required markedly higher exposures. Functionally, ginkgetin reduced clonogenic survival, inhibited migration, and shifted EMT features toward an epithelial phenotype. Apoptosis increased in parallel, accompanied by a pro-apoptotic protein signature. Multi-omics and network analyses converged on PI3K–Akt signaling, and experimental validation showed that ginkgetin primarily dampened pathway output by reducing PI3K/AKT/mTOR phosphorylation rather than total protein abundance. Insulin-mediated reactivation partially reversed phosphorylation suppression and attenuated apoptosis-related shifts, supporting a functional link between axis inactivation and apoptotic tendency. STEAP2 was consistently downregulated after treatment, and STEAP2 overexpression partially counteracted apoptosis-associated changes.

**Conclusion:**

These findings support a coherent “phenotype–pathway–node” model in which ginkgetin inhibits malignant phenotypes and promotes apoptosis in bladder cancer cells, associated with reduced PI3K/AKT/mTOR activity and STEAP2 downregulation. The PI3K/AKT/mTOR axis and STEAP2 emerge as testable mechanistic entry points for further translational validation.

## Introduction

Bladder cancer is one of the most prevalent malignancies of the urinary system worldwide, ranking among the common cancers overall and imposing a disproportionately higher burden on men. In the European Union, age-standardized incidence rates show marked sex differences and substantial geographic heterogeneity, underscoring that bladder cancer remains a major public health challenge [1]. In high-incidence countries such as the United States, the annual number of newly diagnosed cases is considerable, and approximately 75% of patients present with non–muscle-invasive bladder cancer (NMIBC) at initial diagnosis; this epidemiologic profile means that clinical management must largely center on long-term surveillance and risk stratification for early, superficial disease [2, 3]. Although NMIBC is generally associated with favorable 5-year survival compared with the poorer outcomes of localized muscle-invasive bladder cancer (MIBC), its clinical course is characterized by frequent recurrence and a meaningful risk of progression, with 5-year recurrence and progression rates remaining substantial and thereby driving both prognosis and healthcare utilization [4, 5]. This issue is particularly pronounced in high-risk NMIBC, where the likelihood of recurrence and evolution to MIBC further increases, making durable strategies to reduce recurrence/progression a key translational priority [6, 7]. Meanwhile, for localized MIBC, contemporary guideline-based care has shifted from a purely local-control paradigm toward multimodal management, with carefully selected patients considered for bladder-preserving approaches and perioperative immunotherapy to improve tumor control while striving to maintain long-term survival and quality of life [8, 9].

Ginkgetin, a natural biflavonoid isolated from Ginkgo biloba leaves, has emerged as a multi-target bioactive molecule with therapeutic development potential. Accumulating evidence consistently indicates broad antitumor activity across diverse models, including suppression of proliferation, induction of cell-cycle arrest and programmed cell death, attenuation of invasion/metastasis and angiogenesis, and modulation of pivotal signaling networks such as JAK/STAT, Wnt/β-catenin, PI3K/AKT, and MAPK, providing a systematic pharmacologic rationale for its anticancer effects [10, 11]. In non–small cell lung cancer, ginkgetin inhibits proliferation and migration of A549 and H1299 cells, an effect closely linked to blockade of aberrant activation of the FAK/STAT3/AKT axis [12]. In breast cancer models, ginkgetin not only restrains tumor growth but also enhances radiosensitivity, supporting its potential value in combination with standard therapies [13, 14]. In hepatocellular carcinoma cell lines, ginkgetin markedly reduces tumor cell viability by inducing cell-cycle arrest and promoting apoptosis, with concordant in vitro and in vivo findings [15, 16]. In addition, ginkgetin suppresses ovarian cancer cell proliferation, triggers apoptosis, and diminishes migratory and invasive capacities, further extending its anticancer spectrum [17]. Beyond oncology, ginkgetin exerts demonstrable anti-inflammatory effects—for example, mitigating smoke-induced airway inflammation—suggesting utility in inflammation-related disorders [18, 19]. Moreover, in models of neurological disease, ginkgetin shows antioxidant and neuroprotective activities and has been proposed as an adjunctive intervention for ischemic injury or neurodegenerative conditions [20].

## Materials and methods

### Network pharmacology analysis

The chemical structure of ginkgetin was obtained from the PubChem database (https://pubchem.ncbi.nlm.nih.gov/). Putative targets of ginkgetin were predicted using SwissTargetPrediction (http://www.swisstargetprediction.ch) and TargetNet (http://targetnet.scbdd.com/). Bladder cancer–associated genes were collected from GeneCards (https://www.genecards.org/), and genes with a relevance score > 0 were retained as candidate disease-related targets. Next, the target sets for ginkgetin and bladder cancer were imported into Venny 2.1.0 (https://bioinfogp.es/tools/venny/index) to identify overlapping genes, which were regarded as potential therapeutic targets of ginkgetin in bladder cancer. These shared targets were then submitted to the STRING database (https://cn.string-db.org/) to construct a protein–protein interaction (PPI) network, with the organism restricted to Homo sapiens. To investigate the functional implications of these targets, Gene Ontology (GO) and Kyoto Encyclopedia of Genes and Genomes (KEGG) pathway enrichment analyses were performed using DAVID Bioinformatics Resources (https://david.ncifcrf.gov/), aiming to provide functional annotation and enrichment evaluation. Finally, significant GO and KEGG terms (*p* < 0.05) were visualized as bar charts using an online plotting tool (http://www.bioinformatics.com.cn/).

### RNA sequencing and analysis

Total RNA was extracted from 5637 bladder cancer cells treated with ginkgetin and vehicle-treated controls. Two groups were included, each with three independent biological replicates (CONTROL: ctrl_1–3; Ginkgetin: Ginkgetin_1–3). Poly(A) + RNA was enriched using oligo(dT) beads, followed by cDNA library construction according to standard Illumina protocols and paired-end sequencing. Raw reads were quality-filtered using Cutadapt to remove adapters and low-quality sequences, and the resulting clean reads were aligned to the human reference genome (Homo sapiens) using HISAT2. Transcript assembly and quantification were performed with StringTie based on reference annotation, generating both gene- and transcript-level expression matrices; downstream analyses primarily used gene-level expression. Expression abundance was reported as FPKM. Differential expression between ginkgetin-treated and control samples was assessed from read count data using DESeq2 (negative binomial model for replicated designs), and P values were adjusted by the Benjamini–Hochberg method. Genes meeting |log2 fold change| ≥ 1 and q < 0.05 were defined as differentially expressed. Sample reproducibility and global transcriptomic separation were evaluated using Pearson correlation and principal component analysis (PCA). Differentially expressed genes were further interpreted using GO and KEGG over-representation enrichment analyses (hypergeometric testing with multiple-testing correction) and GSEA to capture pathway-level changes without relying on a preset DEG cutoff.

### Molecular docking and molecular dynamics simulation

Molecular docking and molecular dynamics (MD) simulations were conducted to evaluate the binding feasibility and stability of ginkgetin (a Ginkgo biflavonoid) to human STEAP2. The STEAP2 protein sequence was retrieved from UniProtKB (Q9NXH1) and a 3D model was generated by SWISS-MODEL, then saved in PDB format. The 3D structure of ginkgetin was obtained from a public chemical database (e.g., PubChem) and prepared in Discovery Studio (energy minimization and format conversion). For docking, STEAP2 was prepared by removing waters and adding hydrogens/charges in PyMOL, and both receptor and ligand were converted to PDBQT format. Docking was performed using AutoDock Vina with a grid covering the entire STEAP2 structure (blind docking), using 50 runs. The optimal binding pose was selected based on the lowest predicted binding energy and pose recurrence, and interactions were visualized with PyMOL and Discovery Studio. The top-ranked STEAP2–ginkgetin complex was subjected to MD simulation using GROMACS 2022 with the AMBER14SB force field for the protein and GAFF2 parameters for the ligand. The complex was solvated in a TIP3P water box with a 1.0 nm buffer, neutralized with counterions, and treated with PME electrostatics (cutoff 1.0 nm). After energy minimization and equilibration, a 100 ns production run was performed at 310 K under NPT conditions. Trajectories were analyzed using GROMACS tools to calculate RMSD, RMSF, hydrogen bonds, radius of gyration, and SASA to assess complex stability.

### Cell culture and chemicals

Human bladder cancer cell lines (5637, T24, HT-1376, and J82) were purchased from Procell Life Science & Technology Co., Ltd. (Wuhan, China). 5637, T24, and J82 cells were maintained in RPMI 1640 medium (BDBIO, China), whereas HT-1376 cells were cultured in MEM/Eagle medium (BDBIO, China). Both media were supplemented with 10% fetal bovine serum (FBS) and 100 U/mL penicillin plus 100 µg/mL streptomycin (Gibco, USA). Cells were incubated at 37 °C in a humidified atmosphere containing 5% CO₂. Ginkgetin (purity, 99.87%) and insulin were purchased from MedChemExpress (Shanghai, China). Ginkgetin was first dissolved in DMSO to prepare a stock solution and was then diluted in culture medium to the indicated working concentrations immediately before use. Vehicle control groups received the same final concentration of DMSO, which was kept constant across all groups. These bladder cancer cell lines were selected as widely used in vitro models with distinct biological backgrounds, allowing us to reduce reliance on a single-cell-line setting and to assess whether the effects of ginkgetin were reproducible across heterogeneous bladder cancer models. SV-HUC-1 cells were included as a non-malignant urothelial control.

### Cell viability assay

Cell viability was assessed using the Cell Counting Kit-8 (CCK-8; Oriscience, Chengdu, China). Briefly, cells were prepared as single-cell suspensions, counted, and seeded into 96-well plates at a density of approximately 5,000 cells per well, with five technical replicates for each condition. After pre-incubation at 37 °C in a humidified atmosphere containing 5% CO₂ to allow cell attachment, the cells were treated with the indicated concentrations of ginkgetin for 24–48 h. Subsequently, 10 µL of CCK-8 reagent was added to each well, and the plates were incubated for an additional 1–4 h before absorbance was measured at 450 nm using a microplate reader. Each experiment was repeated at least three times independently. For subsequent mechanistic experiments, concentrations below or close to the IC50 were selected to minimize excessive nonspecific cytotoxicity and secondary changes caused by extensive cell death, while preserving detectable signaling and transcriptional responses to ginkgetin. These doses were sufficient to produce reproducible phenotypic and molecular effects in bladder cancer cells. Because 5637 and HT-1376 cells differed in their sensitivity to ginkgetin in the CCK-8 assay, the concentrations used in downstream experiments were determined separately for each cell line on the basis of their respective IC50 ranges and preliminary dose–response results, so as to maintain measurable biological effects while avoiding excessive nonspecific cytotoxicity.

### Flow cytometric analysis of apoptosis

Equal numbers of 5637 and HT-1376 cells were seeded into 6-well plates. After attachment, 5637 cells were treated with ginkgetin at 0, 1, and 1.5 µM for 24 h, while HT-1376 cells were treated with 0, 1, and 2 µM ginkgetin for 24 h. Following treatment, cells were harvested by trypsinization, centrifuged at 850 × g for 5 min, and the supernatant was discarded. Cell pellets were washed twice with phosphate-buffered saline (PBS) and centrifuged under the same conditions to remove residual supernatant. Cells were then resuspended in 500 µL binding buffer, followed by the addition of 5 µL Annexin V and 5 µL propidium iodide (PI). After gentle mixing, samples were incubated for 20 min at room temperature in the dark and analyzed using a CytoFLEX flow cytometer (Beckman Coulter).

### Colony formation assay

5637 and HT-1376 cells were seeded into 6-well plates at a density of 1 × 10³ cells per well and assigned to three experimental groups. After allowing sufficient time for attachment, 5637 cells were treated with ginkgetin at 0, 1, and 1.5 µM, whereas HT-1376 cells were treated with 0, 1, and 2 µM ginkgetin, for 24 h. The medium was then replaced with fresh complete medium, and cells were cultured for an additional 10 days to allow colony formation. At the end of the incubation period, colonies were fixed with 4% paraformaldehyde (Merck, Shanghai, China) for 1 h and stained with 0.1% crystal violet for 30 min. Plates were thoroughly rinsed with double-distilled water, air-dried completely, and colonies were then imaged. Colonies were counted manually from stained plates. A colony was defined as a cluster containing at least 50 cells. Colony numbers from each well were recorded and used for statistical analysis.

### Wound-healing (scratch) assay

Cells in the logarithmic growth phase were resuspended in the appropriate culture medium and seeded into 6-well plates at a density that allowed the monolayer to reach optimal confluence the next day. To ensure imaging at the same field over time, parallel reference lines (0.5–1.0 cm apart) were drawn on the underside of each well with a marker pen. A straight scratch was then generated across the cell monolayer using a sterile pipette tip, and images were captured immediately (0 h). Cells were subsequently maintained in serum-free medium for 24 h, after which images were acquired again at the same locations to document cell migration.

### Western blot analysis

5637 and HT-1376 cells were seeded into 6-well plates at the same density and incubated at 37 °C in a humidified atmosphere containing 5% CO₂ for 24 h. Cells were then treated with the indicated concentrations of ginkgetin for 48 h. After treatment, cells were washed twice with phosphate-buffered saline (PBS) and lysed on ice for 30 min in cold RIPA buffer (Solarbio, China) supplemented with protease and phosphatase inhibitors. Lysates were collected and centrifuged at 12,000 × g for 10 min at 4 °C, and the supernatants were carefully transferred. Protein concentrations were determined using a BCA assay kit (Solarbio, China), and samples were normalized with 1× loading buffer. Equal amounts of protein were separated by 10% SDS–PAGE and transferred onto PVDF membranes (Millipore, USA). Membranes were blocked for 1 h at room temperature in 1× TBST containing 5% nonfat milk, followed by overnight incubation at 4 °C with the following primary antibodies: GAPDH (1:8000; Hu’an Biotech, Zhejiang), Bcl-2 (1:2000; Hu’an Biotech, Zhejiang), Bax (1:20000; Hu’an Biotech, Zhejiang), cleaved caspase-3 (1:1000; Cell Signaling Technology), PI3K (1:2000; Hu’an Biotech, Zhejiang), p-PI3K (1:1000; Hu’an Biotech, Zhejiang), AKT (1:2000; Hu’an Biotech, Zhejiang), p-AKT (1:5000; Hu’an Biotech, Zhejiang), mTOR (1:5000; Hu’an Biotech, Zhejiang), p-mTOR (1:5000; Hu’an Biotech, Zhejiang), E-cadherin (1:5000; Hu’an Biotech, Zhejiang), and N-cadherin (1:5000; Hu’an Biotech, Zhejiang). Membranes were washed three times with 1× TBST (10 min each) and then incubated with HRP-conjugated secondary antibodies (1:10,000; Hu’an Biotech, Zhejiang) for 1 h at room temperature. After three additional washes with TBST (10 min each), signals were developed using an ECL detection reagent (Abbkine, Wuhan, China) and visualized with a Bio-Rad imaging system. Western blot images shown are representative of at least three independent experiments.

### Insulin co-treatment and pathway reactivation assay

To assess whether inhibition of the PI3K/AKT/mTOR pathway by ginkgetin could be functionally reversed, an insulin-mediated pathway reactivation assay was performed. Cells were treated with ginkgetin at the indicated concentrations (1.5 or 2 µM), either alone or together with insulin (10 nM), for 48 h. Insulin was added at the same time as ginkgetin to test whether pathway activation could attenuate the inhibitory effects of ginkgetin during the same treatment period. Four experimental conditions were included: untreated control, insulin alone, ginkgetin alone, and ginkgetin plus insulin. Following treatment, cells were harvested and lysed for protein extraction. The activation status of the PI3K/AKT/mTOR pathway was evaluated by western blotting using antibodies against phosphorylated and total forms of PI3K, AKT, and mTOR, with GAPDH serving as a loading control. In parallel, apoptosis-related proteins Bax and Bcl-2 were examined to assess downstream functional consequences of pathway modulation. Densitometric analyses were performed using ImageJ software. Phosphorylated protein levels were normalized to their corresponding total protein levels, and apoptosis-related proteins were normalized to GAPDH. All experiments were conducted with at least three independent replicates.

### Plasmid transfection and overexpression

For STEAP2 overexpression, a STEAP2 expression plasmid was obtained from GenePharma (Shanghai, China). Transfection was performed according to the manufacturer’s instructions using Lipofectamine 2000 (Thermo Fisher Scientific, USA). Briefly, the plasmid DNA was mixed with an appropriate volume of Lipofectamine 2000 and incubated at room temperature for 20 min to allow complex formation. The DNA–lipid complexes were then added dropwise to cells seeded in 6-well plates. Each well was supplemented with MEM medium (BDBIO, China), and cells were incubated under standard culture conditions for 8 h. The transfection medium was subsequently removed and replaced with complete medium, followed by an additional 24–48 h incubation to allow sufficient expression of the target protein.

### RNA extraction and RT–qPCR

Total RNA was extracted from cultured cells using the Orizen kit (Oriscience, Chengdu, China) according to the manufacturer’s instructions. Reverse transcription was carried out at 37 °C for 20 min. Quantitative real-time PCR was performed using SYBR Green (Tsingke Biotechnology, Beijing, China). GAPDH served as the reference gene, and relative mRNA expression was calculated using the 2^-ΔΔCt method. Primer sequences are listed in Table 1.

**Table 1:** Primer sequences used for qRT-PCR

Gene	Forward primer (5’–3’)	Reverse primer (5’–3’)
GAPDH	AGATCCCTCCAAAATCAAGTGG	GGCAGAGATGATGACCCTTTT
HERC3	TGTTGGGGATATTGGTCTCTGG	CCCTTGGTGTTCAAACCACAT
HIGD1B	ACCTGACGACGAAGACTGTG	CGGTAAATCCTGTATGCTGCTAC
GBP6	ATGGAATCTGGACCCAAAATGTT	GCTGGTTCACCAATAGCTGCT
STEAP2	GGTCACTGTAGGTGTGATTGG	ACCACATGATAGCCGCATCTAA
RHCG	GGTTCCACTTCTTACAAGACCG	GGGCTGACTTTACCCAGAACT
NRIP2	GTTCCTGCACAAGGACTCGG	AGGCGTCTTTGGATCACA
SPATA25	CTACTTCAGGACTCCACAAACTC	CACCACTGGCTCTACAGGACT
ANKRD1	AGTAGAGGAACTGGTCACTGG	TGTTTCTCGCTTTTCCACTGTTC
ZNF572	TGATTCCTATGAGAGTGATGGCA	TGGACACAGGCATTGGTATGT
FAM220A	GAGGTGACTCGGACAAACTATCA	CCACAGGCTTATTCATCCAGGA
BCL-2	GGAGGATTGTGGCCTTCTTTG	AGACAGCCAGGAGAAATCAAACA
BAX	CGGGTTGTCGCCCTTTTCTA	GAGGAAGTCCAATGTCCAGCC

### Statistical analysis

Data are presented as mean ± standard deviation (SD) from at least three independent biological replicates. Statistical analyses were performed using GraphPad Prism software. Comparisons between two groups were conducted using Student’s t-test, while multiple-group comparisons were performed using one-way analysis of variance (ANOVA) followed by appropriate post hoc tests. A P value < 0.05 was considered statistically significant.

## Results

### Ginkgetin suppresses bladder cancer cell viability while exerting minimal effects on normal urothelial cells

**Fig. 1:**
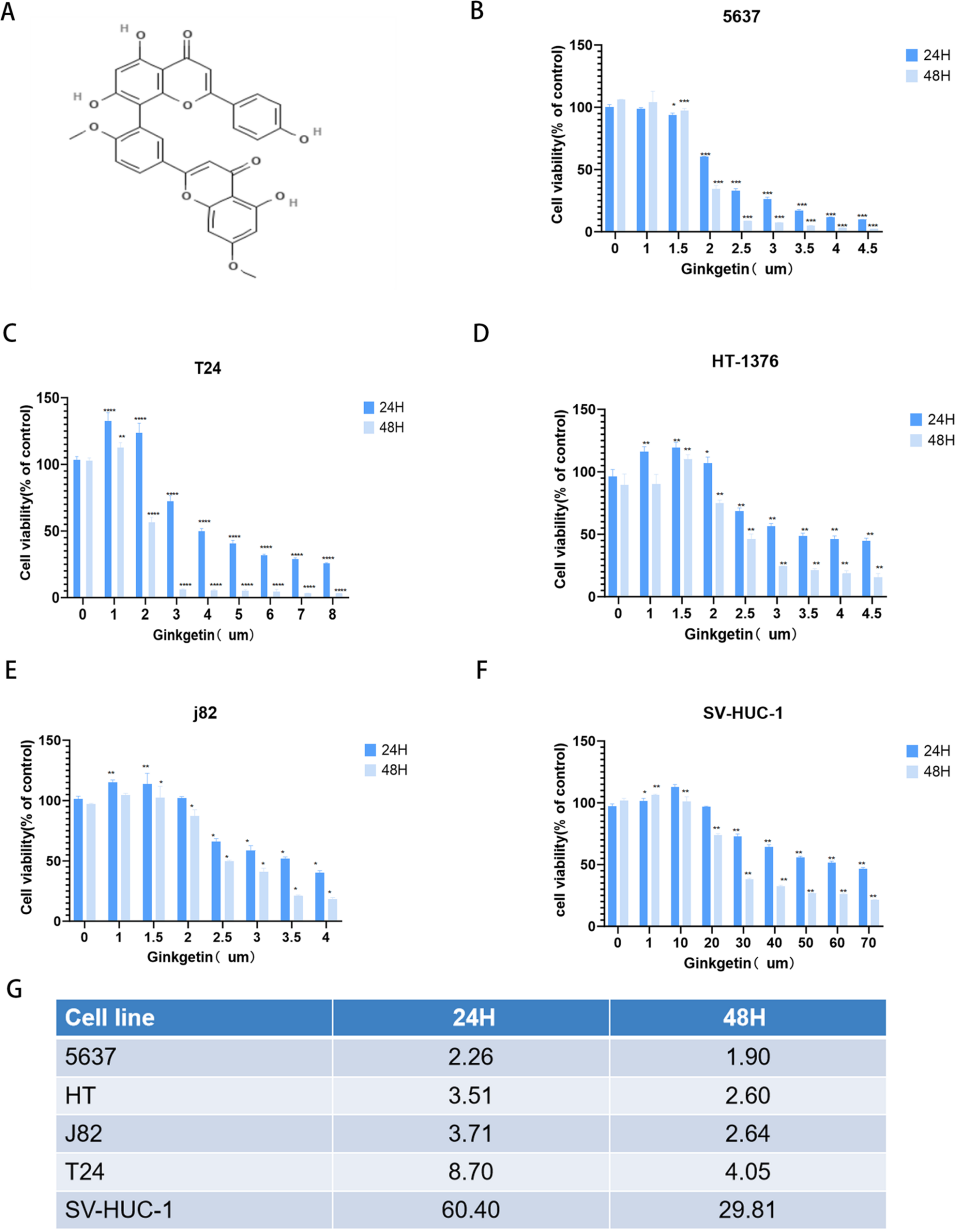
Cytotoxic effects of ginkgetin on bladder cancer cells. **A** Chemical structure of ginkgetin. **B** Cell viability of 5637 bladder cancer cells treated with increasing concentrations of ginkgetin for 24 h and 48 h, as assessed by CCK-8 assay. **C** Cell viability of T24 bladder cancer cells following treatment with ginkgetin at the indicated concentrations for 24 h and 48 h. **D** Cell viability of HT-1376 bladder cancer cells exposed to ginkgetin for 24 h and 48 h. **E** Cell viability of J82 bladder cancer cells treated with various concentrations of ginkgetin for 24 h and 48 h. **F** Cell viability of the normal urothelial cell line SV-HUC-1 after ginkgetin treatment, indicating selective cytotoxicity toward cancer cells. **G** IC₅₀ values of ginkgetin in bladder cancer cell lines and SV-HUC-1 cells at 24 h and 48 h

To evaluate the antitumor activity of ginkgetin, we first obtained its chemical structure from PubChem (Fig. 1A). Four bladder cancer cell lines (5637, T24, HT-1376, and J82) were then exposed to increasing concentrations of ginkgetin, and cell viability was assessed at 24 h and 48 h. Ginkgetin consistently reduced viability in all four cancer cell lines in a clear dose- and time-dependent manner: viability progressively declined with increasing drug concentrations, and the inhibitory effect was generally more pronounced at 48 h than at 24 h (Fig. 1B–E). Sensitivity to ginkgetin varied across cell lines, with 5637 cells showing the strongest response, whereas T24 cells were comparatively less sensitive. Under the same experimental conditions, normal urothelial SV-HUC-1 cells required substantially higher concentrations to exhibit a measurable decrease in viability (Fig. 1F). To quantify these differences, IC₅₀ values were calculated and summarized. At 24 h, the IC₅₀ values for 5637, HT-1376, J82, and T24 cells were 2.26, 3.51, 3.71, and 8.70 µM, respectively, and these values decreased to 1.90, 2.60, 2.64, and 4.05 µM at 48 h. In contrast, the IC₅₀ values for SV-HUC-1 cells were 60.40 µM at 24 h and 29.81 µM at 48 h (Fig. 1G). Collectively, these data indicate that ginkgetin exerts robust growth-inhibitory effects on bladder cancer cells at low micromolar concentrations with enhanced efficacy upon longer exposure, while normal urothelial cells display a markedly higher inhibitory threshold, suggesting a degree of tumor-selective activity.

### Integrated network pharmacology and transcriptomics implicate PI3K/AKT/mTOR signaling in ginkgetin-induced apoptosis in bladder cancer cells

**Fig. 2:**
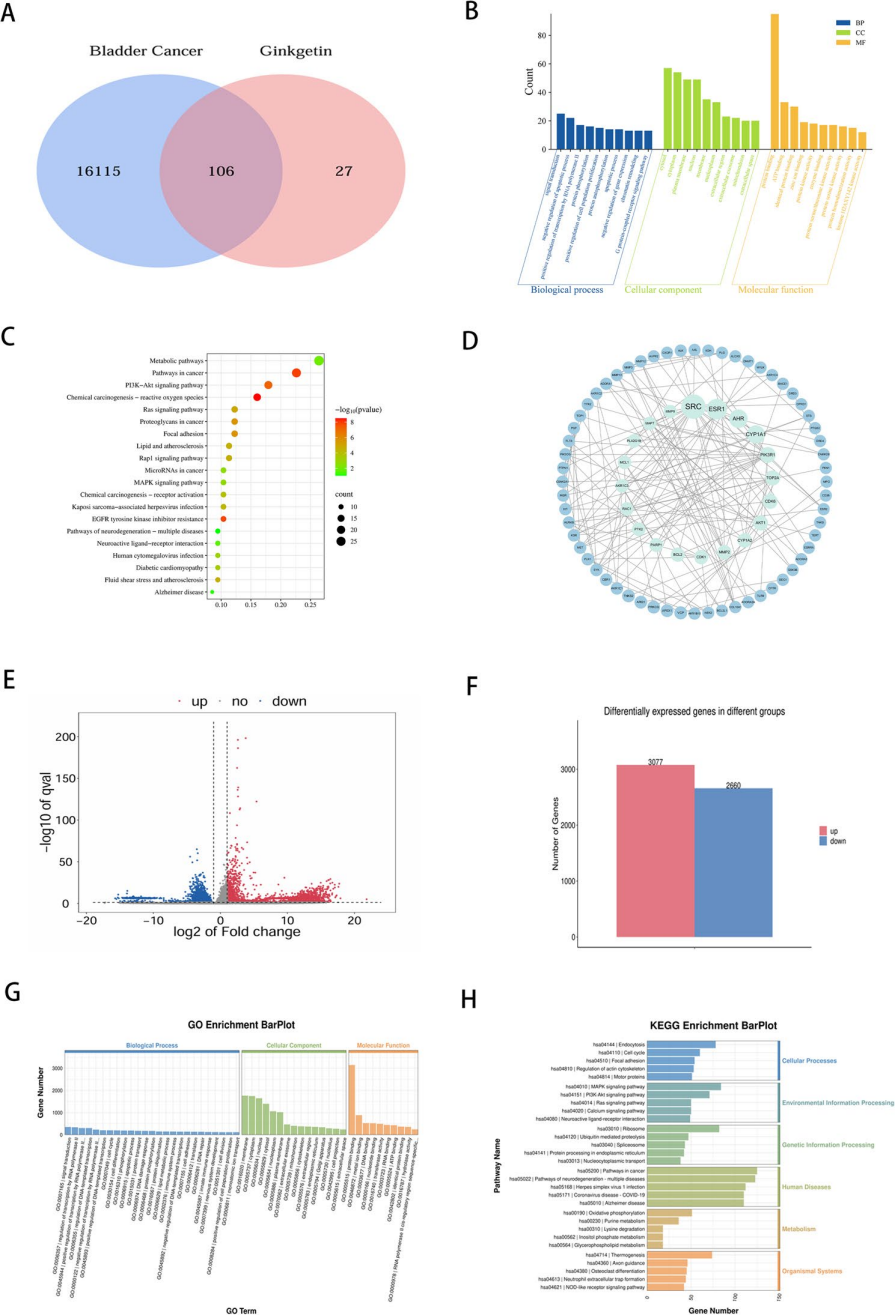
Identification of ginkgetin-related targets and pathway enrichment. **A** Venn diagram showing the overlap between bladder cancer–associated genes and predicted molecular targets of ginkgetin. **B** Gene Ontology enrichment analysis of overlapping genes, categorized into biological process, cellular component, and molecular function. **C** KEGG pathway enrichment analysis of candidate targets, highlighting cancer-related signaling pathways. **D** Protein–protein interaction network of overlapping genes constructed using the STRING database and visualized with Cytoscape. **E** Volcano plot illustrating differentially expressed genes between control and ginkgetin-treated bladder cancer cells. **F** Bar chart showing the numbers of upregulated and downregulated genes following ginkgetin treatment. **G** GO enrichment bar plot of differentially expressed genes. **H** KEGG pathway enrichment bar plot of differentially expressed genes

Network pharmacology analysis revealed a clear overlap between predicted ginkgetin targets and bladder cancer–associated genes, yielding 106 shared candidate targets (Fig. 2A). Gene Ontology (GO) enrichment of these targets indicated that the dominant biological processes were related to transcriptional regulation (including DNA-templated and RNA polymerase II promoter–associated terms), positive/negative regulation of apoptosis, responses to drugs and hypoxic stress, and cell-cycle arrest. At the cellular component level, targets were mainly enriched in the cytoplasm, nucleus, and structures associated with cell junctions and membrane rafts, whereas molecular function terms were dominated by protein binding, ATP binding, zinc ion binding, and interactions with kinases/phosphatases (Fig. 2B). KEGG pathway enrichment further suggested that these targets were concentrated in cancer-relevant signaling programs, prominently including the PI3K–Akt signaling pathway, together with Pathways in cancer, Ras signaling pathway, Proteoglycans in cancer, and Focal adhesion—networks closely linked to proliferation/survival and adhesion/motility (Fig. 2C). Construction of the protein–protein interaction (PPI) network showed dense connectivity, with SRC, ESR1, AHR, PIK3R1, and AKT1 emerging as central hub nodes, supporting a key regulatory position for the PI3K/AKT axis (Fig. 2D).

Transcriptome sequencing performed in parallel provided an independent line of evidence. The volcano plot demonstrated widespread differential expression between the ginkgetin-treated and control groups (Fig. 2E), comprising 3,077 upregulated and 2,660 downregulated genes (Fig. 2F). GO enrichment of differentially expressed genes suggested coordinated functional shifts across BP/CC/MF categories (Fig. 2G), and KEGG analysis again highlighted PI3K–Akt signaling, accompanied by changes in Cell cycle, Focal adhesion, Regulation of actin cytoskeleton, MAPK signaling pathway, and Ras signaling pathway (Fig. 2H). Taken together, these two datasets converge on PI3K/AKT-related signaling at the levels of target prediction, pathway enrichment, and transcriptional response, providing a mechanistic rationale to prioritize the PI3K/AKT/mTOR axis for subsequent validation in ginkgetin-induced apoptosis of bladder cancer cells. The transcriptomic results were then integrated with the network pharmacology analysis to identify convergent pathways and prioritize candidate regulatory nodes for subsequent experimental validation and in silico structural analysis.

### Molecular dynamics analysis of the STEAP2–ginkgetin complex

**Fig. 3:**
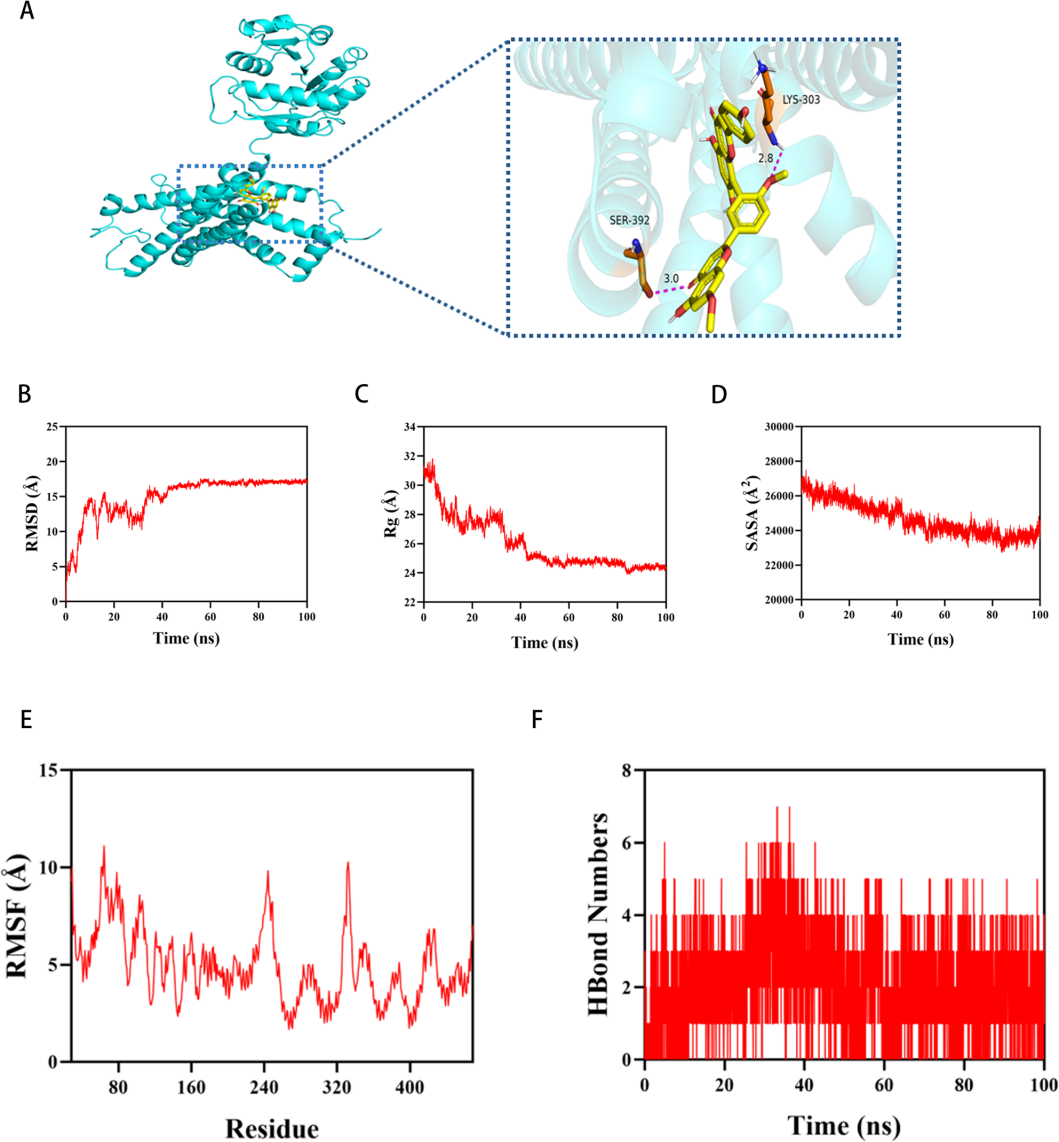
Molecular docking and dynamics simulation of ginkgetin binding to STEAP2 (**A**) Predicted binding conformation of ginkgetin within the STEAP2 protein, highlighting key interacting amino acid residues. **B** Root mean square deviation (RMSD) of the STEAP2–ginkgetin complex during the 100-ns molecular dynamics simulation. **C** Radius of gyration (Rg) of the STEAP2–ginkgetin complex over the simulation time, reflecting structural compactness. **D** Solvent-accessible surface area (SASA) changes of the complex during the simulation. **E** Root mean square fluctuation (RMSF) of individual STEAP2 residues, indicating local flexibility. **F** Number of hydrogen bonds formed between ginkgetin and STEAP2 throughout the simulation

As shown in Fig. 3A, molecular docking revealed that ginkgetin was accommodated within a cavity of STEAP2, forming stable polar interactions with key residues, including LYS303 and SER392, with hydrogen-bond distances of approximately 2.8–3.0 Å. These interactions provided a structural basis for subsequent dynamic simulations. To evaluate the dynamic behavior of the STEAP2–ginkgetin complex, a 100 ns molecular dynamics simulation was performed. Root-mean-square deviation (RMSD) analysis (Fig. 3B) showed relatively large fluctuations during the first ~ 40 ns, likely reflecting an initial structural adjustment of the STEAP2–ginkgetin complex from the starting docked conformation. Thereafter, the RMSD gradually stabilized and plateaued at around ~ 17 Å without persistent drift, suggesting that the complex underwent an early global conformational rearrangement and subsequently reached a quasi-equilibrated state. The radius of gyration (Rg) decreased progressively throughout the simulation (Fig. 3C), suggesting that the STEAP2–ginkgetin complex gradually adopted a more compact overall conformation. In parallel, the solvent-accessible surface area (SASA) exhibited a continuous downward trend (Fig. 3D), indicating reduced solvent exposure during equilibration. This reduction in SASA may also reflect decreased accessibility of parts of the binding interface, consistent with tighter packing of the complex rather than progressive dissociation. Together, these results suggest that ligand binding was accompanied by global structural compaction and reduced solvent exposure, consistent with stabilization of the protein–ligand complex. Analysis of residue-level flexibility using RMSF (Fig. 3E) showed that most residues fluctuated below ~ 8 Å, with higher flexibility confined to a limited number of regions, consistent with localized conformational mobility rather than overall structural instability. Hydrogen-bond analysis (Fig. 3F) demonstrated that 0–7 hydrogen bonds were intermittently formed during the simulation, with approximately 2–4 hydrogen bonds maintained for the majority of the trajectory, supporting the presence of persistent polar interactions between ginkgetin and STEAP2. Collectively, these molecular dynamics results indicate that the STEAP2–ginkgetin complex undergoes an initial conformational adjustment followed by stabilization, characterized by global rearrangement, increased compactness, and sustained intermolecular interactions.

### Ginkgetin promotes apoptosis and impairs clonogenic capacity in bladder cancer cells

**Fig. 4:**
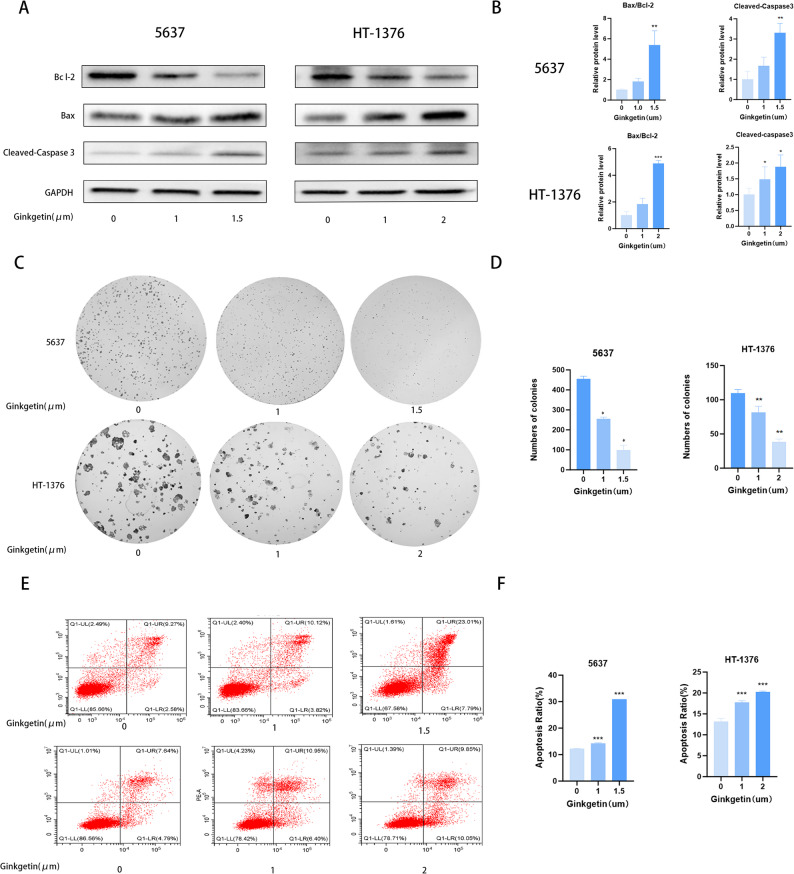
Ginkgetin induces apoptosis in bladder cancer cells. **A** Representative Western blot images showing Bcl-2, Bax, and cleaved caspase-3 expression in 5637 and HT-1376 cells treated with ginkgetin. **B** Quantitative analysis of Bax/Bcl-2 ratio and cleaved caspase-3 protein levels. **C** Representative colony formation assay images of bladder cancer cells treated with ginkgetin. **D** Quantification of colony numbers following ginkgetin treatment. **E** Flow cytometric analysis of apoptosis in bladder cancer cells treated with ginkgetin. **F** Statistical analysis of apoptotic cell percentages

We next examined apoptosis-related molecular events and long-term proliferative potential in 5637 and HT-1376 bladder cancer cells following ginkgetin exposure. Immunoblotting showed that, across increasing concentrations of ginkgetin (5637: 0, 1, 1.5 µM; HT-1376: 0, 1, 2 µM), the anti-apoptotic protein Bcl-2 decreased stepwise, whereas Bax and cleaved caspase-3 increased, consistent with activation of apoptosis-associated signaling (Fig. 4A). Densitometric quantification corroborated these changes, showing a significant dose-dependent rise in the Bax/Bcl-2 ratio and in relative cleaved caspase-3 abundance, with the most pronounced shifts observed at higher doses (Fig. 4B).

Functionally, clonogenic assays demonstrated that ginkgetin markedly reduced colony-forming ability in both cell lines in a dose-dependent manner: compared with controls, even the lower dose reduced colony density, and the inhibitory effect became more evident at higher doses (Fig. 4C). Colony counting further quantified this trend, revealing a significant decline in colony numbers with increasing ginkgetin concentration in both 5637 and HT-1376 cells (Fig. 4D). To directly assess apoptosis at the cellular level, Annexin V/PI flow cytometry was performed. Representative dot plots showed an expansion of the Annexin V–positive population as the drug concentration increased (Fig. 4E), and quantitative analysis confirmed that total apoptotic rates rose significantly in both cell lines, with a greater increment in the high-dose groups (Fig. 4F). Together, the protein-level changes, clonogenic suppression, and flow-cytometric apoptosis readouts converge to indicate that ginkgetin enhances apoptosis while weakening long-term clonogenic survival in 5637 and HT-1376 cells.

### Ginkgetin suppresses bladder cancer cell migration and reverses EMT-associated features

**Fig. 5:**
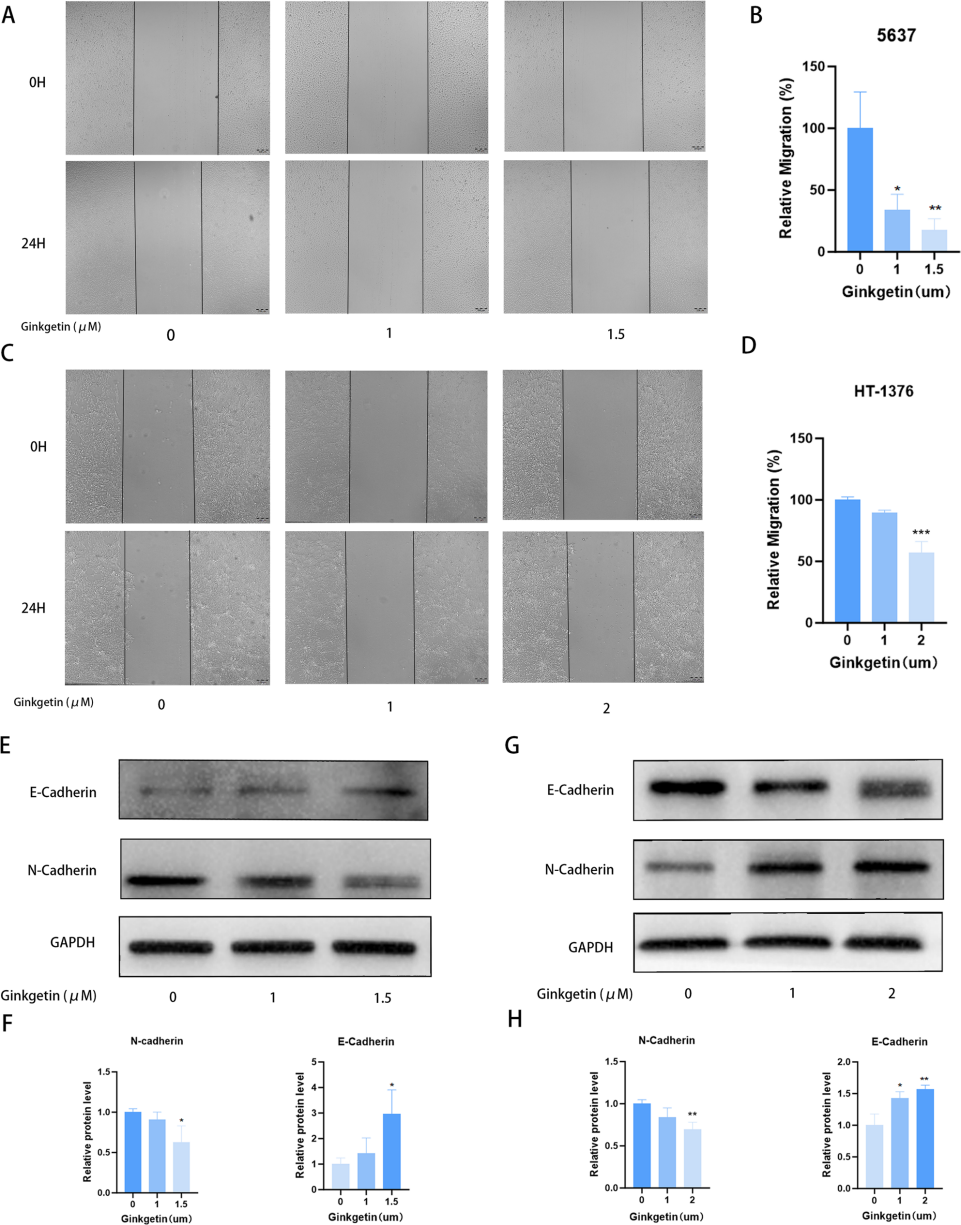
Ginkgetin inhibits migration and epithelial–mesenchymal transition. **A** Representative wound-healing assay images of 5637 cells at 0 h and 24 h after ginkgetin treatment. **B** Quantification of relative migration rate of 5637 cells. **C** Representative wound-healing assay images of HT-1376 cells following ginkgetin treatment. **D** Quantitative analysis of migration in HT-1376 cells. **E** Western blot analysis of E-cadherin and N-cadherin expression in 5637 cells treated with ginkgetin. **F** Densitometric quantification of EMT-related protein expression in 5637 cells. **G** Western blot analysis of EMT markers in HT-1376 cells following ginkgetin treatment. **H** Quantification of E-cadherin and N-cadherin protein levels in HT-1376 cells

To evaluate the impact of ginkgetin on migratory capacity, wound-healing assays were performed in 5637 and HT-1376 cells. In 5637 cells, treatment with ginkgetin (0, 1, 1.5 µM) for 24 h resulted in visibly delayed wound closure relative to the control, and the extent of closure decreased further as the dose increased (Fig. 5A). Quantification confirmed a dose-dependent reduction in relative migration (Fig. 5B). A comparable pattern was observed in HT-1376 cells: control cultures exhibited more pronounced closure at 24 h, whereas ginkgetin (0, 1, 2 µM) restricted wound healing to varying degrees, with the strongest inhibition at the highest dose (Fig. 5C), and statistical analysis verified that relative migration decreased significantly with dose escalation (Fig. 5D).

Given the close relationship between migratory behavior and epithelial–mesenchymal transition (EMT), we further assessed EMT marker expression. In 5637 cells, immunoblotting revealed increased E-cadherin and decreased N-cadherin following ginkgetin treatment (Fig. 5E), and densitometric analysis supported these changes with a clear dose-responsive trend (Figure. 5 F). In HT-1376 cells, ginkgetin similarly increased E-cadherin while reducing N-cadherin (Fig. 5G), and quantification confirmed statistically significant, dose-progressive shifts (Fig. 5H). Overall, the wound-healing phenotypes and EMT marker remodeling consistently indicate that ginkgetin suppresses migration in both 5637 and HT-1376 cells while steering EMT-associated features toward a more epithelial-like state.

### Ginkgetin inhibits PI3K/AKT/mTOR signaling, and insulin activation partially rescues this effect

**Fig. 6:**
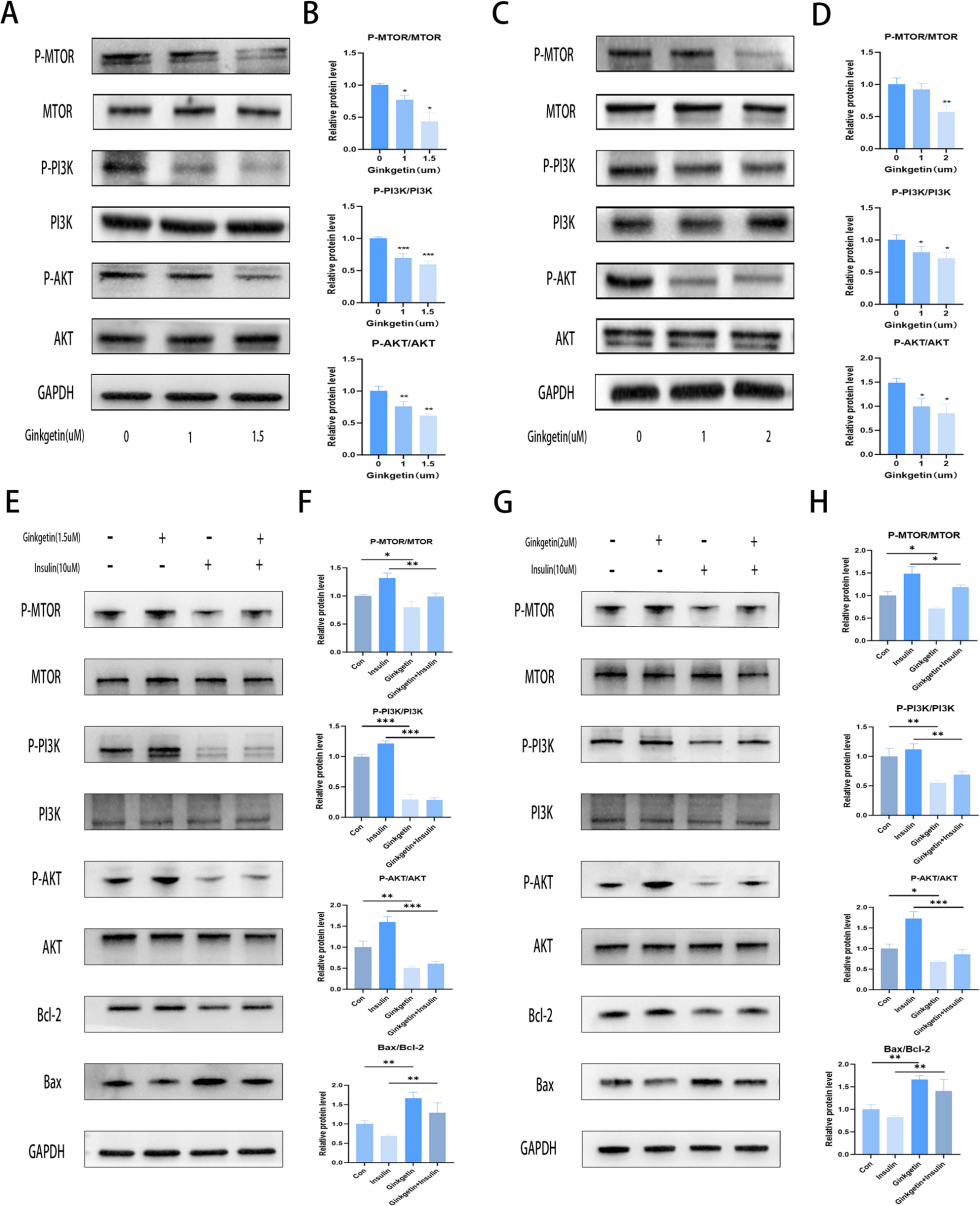
Ginkgetin suppresses PI3K/AKT/mTOR signaling. **A** Western blot analysis of phosphorylated and total PI3K, AKT, and mTOR proteins in 5637 cells treated with ginkgetin. **B** Quantitative analysis of p-mTOR/mTOR, p-PI3K/PI3K, and p-AKT/AKT ratios in 5637 cells. **C** Western blot analysis of phosphorylated and total PI3K, AKT, and mTOR proteins in HT-1376 cells treated with ginkgetin. **D** Quantitative analysis of p-mTOR/mTOR, p-PI3K/PI3K, and p-AKT/AKT ratios in HT-1376 cells. **E** Effects of insulin stimulation on PI3K/AKT/mTOR signaling in 5637 cells in the presence or absence of ginkgetin. **F** Quantitative analysis of pathway activation in 5637 cells under insulin and ginkgetin co-treatment. **G** Western blot analysis of PI3K/AKT/mTOR signaling in HT-1376 cells under insulin and ginkgetin co-treatment. **H** Quantitative analysis of pathway activation in HT-1376 cells under combined treatment

We then interrogated the activation status of the PI3K/AKT/mTOR axis by assessing phosphorylation levels. In 5637 cells, immunoblotting showed a progressive reduction in p-PI3K, p-AKT, and p-mTOR signals with increasing ginkgetin concentration, whereas total PI3K, AKT, and mTOR levels remained largely stable, suggesting that ginkgetin primarily dampens pathway activation rather than reducing protein abundance (Fig. 6A). Consistently, densitometric ratios (p-PI3K/PI3K, p-AKT/AKT, and p-mTOR/mTOR) declined in a dose-dependent manner with statistical significance (Fig. 6B). The same pattern was reproduced in HT-1376 cells, where ginkgetin markedly lowered phosphorylation across the PI3K/AKT/mTOR axis with minimal changes in total protein expression (Fig. 6C), and ratio-based quantification confirmed significant inhibition (Fig. 6D).

To functionally probe the relationship between pathway activation and drug-associated phenotypes, insulin was used as an upstream stimulus to reactivate PI3K/AKT/mTOR signaling. In 5637 cells, insulin increased p-PI3K, p-AKT, and p-mTOR, whereas ginkgetin alone suppressed these phosphorylation signals and was accompanied by a shift in apoptosis-related protein expression toward a pro-apoptotic direction. When insulin and ginkgetin were combined, insulin-driven reactivation partially counteracted the ginkgetin-induced decrease in phosphorylation, and the accompanying apoptosis-related changes showed a partial reversal (Fig. 6E). Statistical analysis further demonstrated significant restoration of p-mTOR/mTOR, p-PI3K/PI3K, and p-AKT/AKT in the combination group compared with the ginkgetin-only group, along with a rebound tendency in apoptosis-associated readouts (Fig. 6F). Similar partial counterbalancing effects were observed in HT-1376 cells, where insulin attenuated the ginkgetin-associated suppression of pathway phosphorylation and the related apoptotic shifts (Fig. 6G), and quantification supported the internal consistency of this “inhibition–reactivation intervention” pattern (Fig. 6H). Taken together, these results indicate that ginkgetin reduces phosphorylation-dependent activation of PI3K/AKT/mTOR signaling in parallel with apoptosis-related molecular changes, and that upstream pathway reactivation by insulin can partially mitigate these effects, supporting a functional involvement of this axis in the action of ginkgetin.

### STEAP2 participates in regulating ginkgetin-induced apoptotic responses in bladder cancer cells

**Fig. 7:**
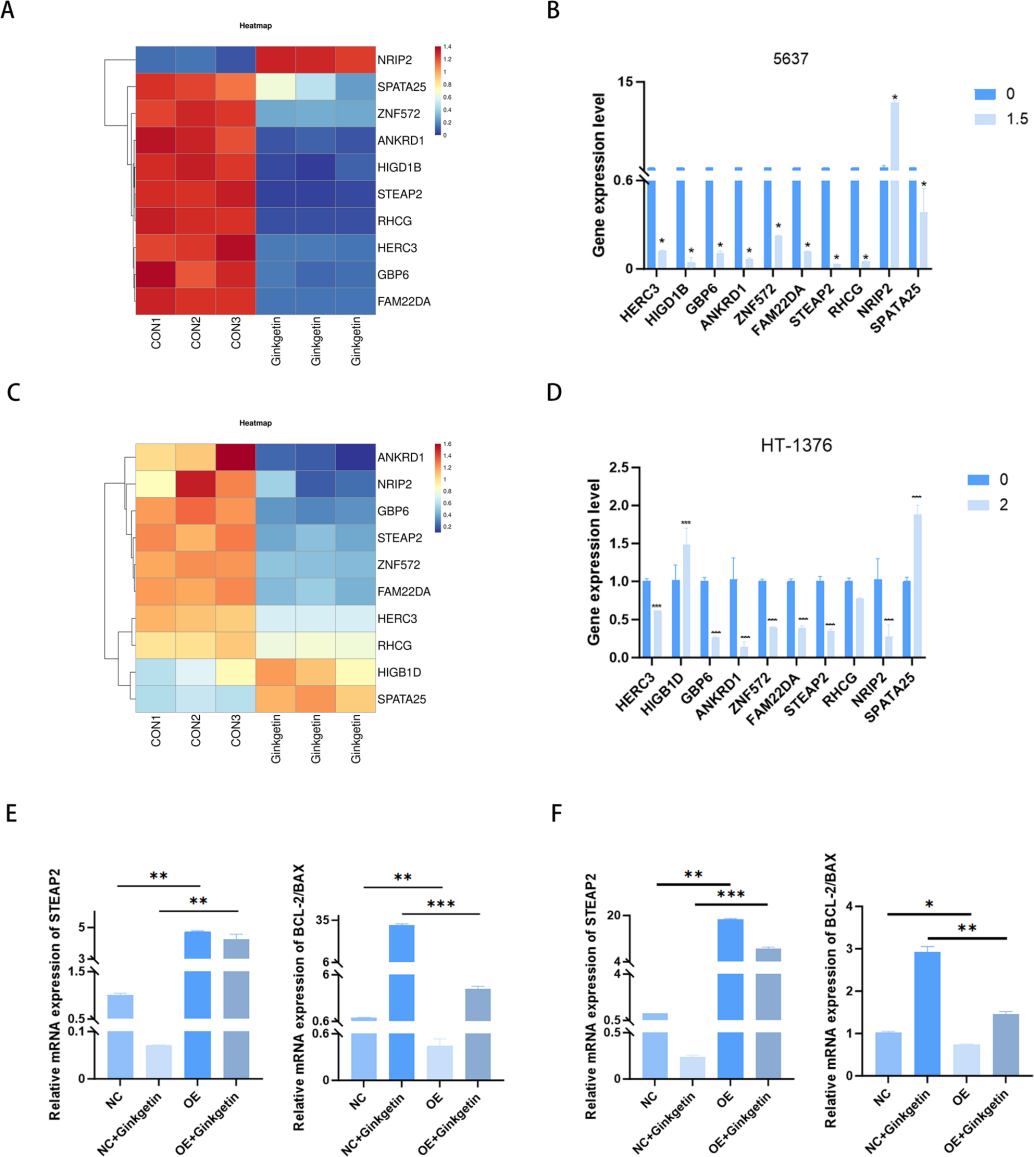
Validation of STEAP2-related gene expression. **A** Heatmap showing differential expression of selected genes in control and ginkgetin-treated 5637 cells. **B** Quantitative analysis of gene expression changes in 5637 cells following ginkgetin treatment. **C** Heatmap illustrating gene expression alterations in HT-1376 cells. **D** Statistical analysis of gene expression levels in HT-1376 cells after ginkgetin treatment. **E** Relative mRNA expression of STEAP2 and apoptosis-related genes under different experimental conditions in 5637 cells. **F** Quantitative analysis of STEAP2 and BCL-2/BAX expression in HT-1376 cells

Unsupervised clustering of the heatmap showed a clear separation between ginkgetin-treated samples and controls at the global expression level, indicating a consistent transcriptomic remodeling after treatment (Fig. 7A). In 5637 cells, qPCR validation of selected candidate differentially expressed genes revealed treatment-associated expression shifts, among which STEAP2 was markedly downregulated following ginkgetin exposure (Fig. 7B). In HT-1376 cells, candidate genes displayed concordant expression changes after treatment (Fig. 7C), and quantitative analysis confirmed that multiple genes—including STEAP2—changed significantly (Fig. 7D).

To further examine the functional relevance of STEAP2 to apoptosis-related phenotypes, 5637 cells were assigned to NC, NC+ginkgetin, STEAP2 overexpression (OE), and OE+ginkgetin groups. Ginkgetin reduced STEAP2 mRNA levels and was accompanied by changes in the BCL-2/BAX ratio. Under STEAP2 overexpression, STEAP2 expression increased markedly, and the magnitude of the ginkgetin-associated changes in STEAP2 and BCL-2/BAX showed partial attenuation in the OE+ginkgetin group compared with ginkgetin alone (Fig. 7E). The same pattern was observed in HT-1376 cells: ginkgetin decreased STEAP2 expression and altered BCL-2/BAX, whereas STEAP2 overexpression partially offset these changes (Fig. 7F).

## Discussion

With translation in mind, we aimed to connect phenotype-level changes induced by ginkgetin with a signaling axis that can be experimentally tested. In vitro, bladder cancer cells consistently responded to low-micromolar ginkgetin, whereas normal urothelial cells required markedly higher exposures to show comparable suppression, suggesting a potential (though still preliminary) therapeutic window. Rather than starting from a preselected “favorite” molecule, we used network pharmacology and transcriptomics to establish directionality; both streams converged on a PI3K–Akt–centered module, which then guided our validation strategy [21]. A set of complementary functional readouts reinforced that ginkgetin does more than reduce short-term viability: apoptosis increased, clonogenic capacity declined, and migration/EMT traits were simultaneously restrained. Importantly, phosphorylation-level inhibition of PI3K/AKT/mTOR, together with partial counteraction after insulin-mediated reactivation, provided an interpretable framework in which dampened survival signaling is coupled to enhanced apoptotic propensity [22]. We further anchored the transcriptional response to an experimentally tractable node, STEAP2: its expression decreased reproducibly in both cell lines, and forced overexpression partially offset apoptosis-related shifts, implying that STEAP2 may help bridge pathway state and apoptotic output. Collectively, our data place a natural product–driven antitumor phenotype, a testable signaling backbone, and a candidate regulatory molecule on a single evidence chain, creating clear entry points for mechanistic closure and future translational work [23].

### A multi-dimensional pharmacological profile of ginkgetin and its positioning in bladder cancer

Ginkgetin is of interest not simply because it shows antitumor activity, but because it often behaves like a network modulator across disease contexts. In cancer models, prior studies indicate that ginkgetin can concurrently affect proliferation and migration/invasion, accompanied by regulation of survival- and metastasis-related pathways such as PI3K/AKT, Wnt/β-catenin, and JAK/STAT, forming a relatively consistent antitumor phenotypic spectrum [24–26]. Beyond monotherapy-like effects, combination potential has also been suggested; in EGFR wild-type NSCLC settings, ginkgetin reportedly enhances cisplatin responses, in part by inducing ferroptosis and suppressing the Nrf2/HO-1 antioxidant axis, thereby weakening cellular tolerance to stress [27]. Evidence outside oncology further highlights its pleiotropy: ginkgetin has been identified as a direct STING-binding molecule that suppresses cGAS–STING signaling and ameliorates systemic inflammation and aging-related phenotypes [28]; transcriptomic and single-cell evidence in metabolic disease models supports improvements in NASH-associated pathology with reprogramming of key cellular populations [29]. In neurological and peripheral nerve repair–related studies, ginkgetin has also been linked to effects on apoptosis, neuroprotection, or cell migration [30–32]. These cross-domain observations support viewing ginkgetin as a regulator of a “survival–stress–inflammation” network, which makes it biologically reasonable—within bladder cancer—to focus on whether suppression of survival signaling lowers the threshold for apoptosis.

### Interpreting our findings through an apoptosis-centered framework

Apoptosis is a tightly programmed cell-death modality executed through caspase cascades; in the mitochondrial pathway, BCL-2 family proteins regulate mitochondrial outer membrane permeabilization, trigger cytochrome c release, and ultimately activate effector caspases [33, 34]. Tumor cells commonly resist apoptosis by maintaining chronic survival signaling, and PI3K/AKT/mTOR is a canonical upstream scaffold that supports metabolism and stress tolerance while raising the threshold for apoptotic initiation; accordingly, its inhibition is widely regarded as a rational pro-apoptotic strategy [35, 36]. Prior evidence already suggests ginkgetin can promote apoptosis—for example, in hepatocellular carcinoma models it reportedly enhances caspase-3 activity and cytochrome c release with increased apoptosis. Our study extends this pro-apoptotic signal into bladder cancer and strengthens mechanistic plausibility by integrating a pathway “inhibition–reactivation” logic: suppression of PI3K/AKT/mTOR phosphorylation coincided with apoptosis-related shifts, whereas insulin-driven reactivation partially attenuated these changes. Together with STEAP2 as an actionable entry point, future work can more directly address how survival-axis inactivation rewires apoptotic sensitivity.

### Rationale for selecting STEAP2 as a mechanistic node

Among transcriptome-derived candidates, STEAP2 was prioritized not merely because of expression change, but because prior literature links it to survival signaling and invasive phenotypes. One study suggests STEAP2 perturbation can influence PI3K/AKT/mTOR-related signaling and contribute to regulation of tumor cell proliferation and metastasis [37]. Another report places STEAP2 within a PI3K/AKT/mTOR-driven EMT program to explain invasion and migration in osteosarcoma cells [38]. In our framework, STEAP2 therefore functions as a plausible bridge between drug-induced transcriptional remodeling and phenotypic outputs. Conceptually, our reasoning follows a clinically familiar sequence: prediction defines pathway direction, experiments verify pathway–phenotype coupling (phosphorylation suppression with partial reversal upon reactivation), and a manipulable node (STEAP2) provides a concrete handle for mechanistic refinement. This positioning turns STEAP2 from a “differential gene” into a testable candidate for genetic strategies aimed at closing the loop.

### Limitations

Several limitations should be noted. First, both network pharmacology and transcriptomic analyses suggested that ginkgetin may affect multiple pathways. Although we focused on the PI3K/AKT/mTOR pathway and obtained supporting functional evidence, other pathways, such as those involved in cell cycle regulation, adhesion and cytoskeletal remodeling, MAPK signaling, and Ras signaling, may also participate in the observed effects, either independently or through cooperative or compensatory interactions. These possibilities require further investigation. Second, although the decrease in STEAP2 expression, together with the rescue effects observed in the overexpression and insulin co-treatment experiments, supports a role for STEAP2-related pathway modulation, additional loss-of-function studies and direct target-binding assays are still needed to clarify the underlying mechanism. Likewise, the molecular docking and molecular dynamics analyses provide only supportive structural evidence and cannot by themselves confirm biologically meaningful target engagement. Therefore, while STEAP2 appears to be a potentially relevant mechanistic node, its biological and translational significance remains to be established, particularly in the absence of in vivo or clinical evidence. Third, our conclusions are mainly based on in vitro models. Important translational issues, including pharmacokinetics, tissue distribution, the actual therapeutic window, and the potential influence of molecular subtype or genetic background on ginkgetin sensitivity, could not be addressed in the present study. Further validation in xenograft or orthotopic models, as well as in patient-derived systems and clinical specimens, will be necessary to better define its translational relevance.

## Conclusion

We assembled a “phenotype–pathway–node” line of evidence to clarify how ginkgetin acts against bladder cancer. At low micromolar concentrations, ginkgetin consistently reduced cell viability and clonogenic survival, slowed migration, and shifted EMT-related features toward a more epithelial state, while apoptosis-associated readouts increased in parallel. Mechanistically, both network pharmacology and transcriptomic profiling converged on the PI3K–Akt signaling network. Experimental validation further suggested that ginkgetin primarily dampens pathway output by lowering the phosphorylation (activation) of the PI3K/AKT/mTOR axis rather than altering total protein abundance. When insulin was used to re-activate this axis, several molecular changes and corresponding phenotypes showed partial rebound, supporting a functional link between pathway inactivation and enhanced apoptotic tendency. In the same context, STEAP2 was consistently downregulated across cell models, and STEAP2 overexpression partially counteracted apoptosis-related changes, implying that STEAP2 may serve as an actionable regulatory node coupling transcriptional reprogramming to apoptotic output. Taken together, our findings support a working model in which ginkgetin promotes apoptosis and suppresses malignant phenotypes in bladder cancer cells, accompanied by STEAP2 downregulation and reduced PI3K/AKT/mTOR pathway activity, offering a testable mechanistic framework for further translational investigation.

## Data Availability

Supporting data for this study are available from the corresponding author on reasonable request.

## Abbreviations

ANOVA: Analysis of variance
BAX: BCL2 associated X protein
BCA: Bicinchoninic acid (assay)
BCL-2: B-cell lymphoma 2
BP: Biological process
CC: Cellular component
CCK-8: Cell Counting Kit-8
cGAS: Cyclic GMP–AMP synthase
cGAS-STING: cGAS–STING signaling pathway
DAVID: Database for Annotation, Visualization and Integrated Discovery
DEG: Differentially expressed gene(s)
ECL: Enhanced chemiluminescence
EGFR: Epidermal growth factor receptor
EMT: Epithelial–mesenchymal transition
FBS: Fetal bovine serum
FPKM: Fragments per kilobase of transcript per million mapped reads
GO: Gene Ontology
GROMACS: GROningen MAchine for Chemical Simulations
GSEA: Gene set enrichment analysis
HISAT2: Hierarchical Indexing for Spliced Alignment of Transcripts 2
IC50: Half-maximal inhibitory concentration
KEGG: Kyoto Encyclopedia of Genes and Genomes
MD: Molecular dynamics
MEM: Minimum Essential Medium
MIBC: Muscle-invasive bladder cancer
NC: Negative control
NMIBC: Non–muscle-invasive bladder cancer
NPT: Constant number of particles, pressure, and temperature (ensemble)
NSCLC: Non–small cell lung cancer
OE: Overexpression
PBS: Phosphate-buffered saline
PCA: Principal component analysis
PCR: Polymerase chain reaction
PDB: Protein Data Bank (format)
PDBQT: PDBQT format (used by AutoDock)
PI: Propidium iodide
PME: Particle mesh Ewald
PPI: Protein–protein interaction
PVDF: Polyvinylidene difluoride
qPCR: Quantitative real-time PCR
RIPA: Radioimmunoprecipitation assay (buffer)
RMSD: Root-mean-square deviation
RMSF: Root-mean-square fluctuation
Rg: Radius of gyration
RPMI: Roswell Park Memorial Institute (medium)
SASA: Solvent-accessible surface area
SDS-PAGE: Sodium dodecyl sulfate–polyacrylamide gel electrophoresis
SD: Standard deviation
STEAP2: Six-transmembrane epithelial antigen of prostate 2
STING: Stimulator of interferon genes
STRING: Search Tool for the Retrieval of Interacting Genes/Proteins
SYBR: SYBR Green dye
TBST: Tris-buffered saline with Tween-20
TIP3P: Three-point transferable intermolecular potential (water model)
V/PI: Annexin V / propidium iodide
